# Prenatal Diagnosis of Bartter Syndrome: Lessons From a Complex Diagnostic Procedure

**DOI:** 10.1002/ccr3.70749

**Published:** 2025-08-27

**Authors:** Athina A. Samara, Paschalis Mousios, Paraskevas Perros, Antonios Koutras, Emmanouil Manolakos, Eleftherios Anastasakis, Chara Skentou, Konstantinos Dafopoulos, Antonios Garas, Sotirios Sotiriou

**Affiliations:** ^1^ Department of Embryology, Faculty of Medicine University of Thessaly Larissa Greece; ^2^ Department of Obstetrics and Gynaecology Alexandra General Hospital Athens Greece; ^3^ Access to Genome‐ATG Clinical Laboratory Genetics Athens Greece; ^4^ Iaso Maternity Hospital Athens Greece; ^5^ Department of Obstetrics and Gynaecology, Medical School University of Ioannina Ioannina Greece; ^6^ Department of Obstetrics and Gynaecology University Hospital of Larissa Larissa Greece

**Keywords:** aldosterone, amnioreduction, Bartter syndrome, polyhydramnios, prenatal testing, renin

## Abstract

Bartter syndrome is an idiopathic condition that may manifest antenatally, characterized by a spectrum of symptoms including maternal polyhydramnios, prematurity, polyuria, hypercalciuria, nephrocalcinosis, normomagnesemia, vomiting, growth retardation, and elevated renal synthesis and urinary excretion of prostaglandins. Herein, we present a case of a 36‐year‐old Caucasian woman (Gravida 1, Para 0) diagnosed with extensive hydramnios at the 27th week of gestation. A decision for decompressive amniocentesis was made, and the amniotic fluid was sent for biochemical testing. The biochemical results from the amniotic fluid were compatible with Bartter syndrome, and the sample was further processed for genetic testing. A week later, the hydramnios reoccurred, and a decision for a second decompression amniocentesis was made. Twelve hours following the procedure, premature membrane rupture occurred, and a female fetus weighing 925 g was delivered via an emergency cesarean section for breech presentation. In conclusion, when a case is diagnosed with idiopathic polyhydramnios, investigating underlying genetic or renal syndromes such as Bartter syndrome using comprehensive diagnostic protocols is essential.

## 1 Introduction

Polyhydramnios refers to the abnormal accumulation of amniotic fluid during pregnancy, occurring in approximately 1%–2% of pregnancies. It can arise due to various factors including maternal diabetes, viral infections, genetic disorders, and conditions such as neuromuscular disorders and maternal hypercalcemia [1]. Bartter syndrome, a rare inherited disorder affecting kidney function, is a common cause of idiopathic polyhydramnios. This syndrome disrupts salt reabsorption in the ascending limb of the loop of Henle, leading to salt wasting, hypokalemia, and metabolic alkalosis [2].

Antenatally, this rare autosomal recessive renal tubular disorder affects the fetus. It involves defective chloride transport in the loop of Henle, leading to fetal polyuria and severe polyhydramnios, thereby increasing the risks of premature delivery and intrauterine growth restriction (IUGR) [3, 4, 5]. The syndrome is characterized by metabolic alkalosis and hypokalemia. Biochemical analysis of the amniotic fluid can aid in antenatal diagnosis, revealing normal sodium and potassium levels but elevated chloride levels [5, 6, 7, 8]. Aldosterone levels in amniotic fluid remain in normal ranges in most Bartter cases [9, 10]. Renin levels in amniotic fluid could be a possible marker for the syndrome; however, because of the need for a specific technical adjustment for measurements in amniotic fluid, there is no data regarding these values [11].

On the other hand, a rare form of X‐linked polyhydramnios with prematurity and a severe but transient form of antenatal Bartter's syndrome has been described, with MAGE‐D2 being essential for fetal renal salt reabsorption, amniotic fluid homeostasis, and the maintenance of pregnancy [12]. In a French cohort [13], MAGE‐D2 mutations explained 9% of cases of antenatal Bartter syndrome and reported that female embryos can be affected too. A finding worth mentioning is the observation that these neonates were not suffering from IUGR, but their growth was at the 94th percentile [13].

Herein, we report an interesting and rare case of a prenatal diagnosis of Bartter syndrome as a cause of extensive polyhydramnios treated with decompressive amniocentesis during the 27th week of gestation. Based on our experience, we aim to raise clinical suspicion in the prenatal diagnosis of the syndrome and highlight the need for intensive monitoring and care of these cases in a tertiary department with an experienced neonatal unit.

### 1.1 Case History/Examination

**FIGURE 1:**
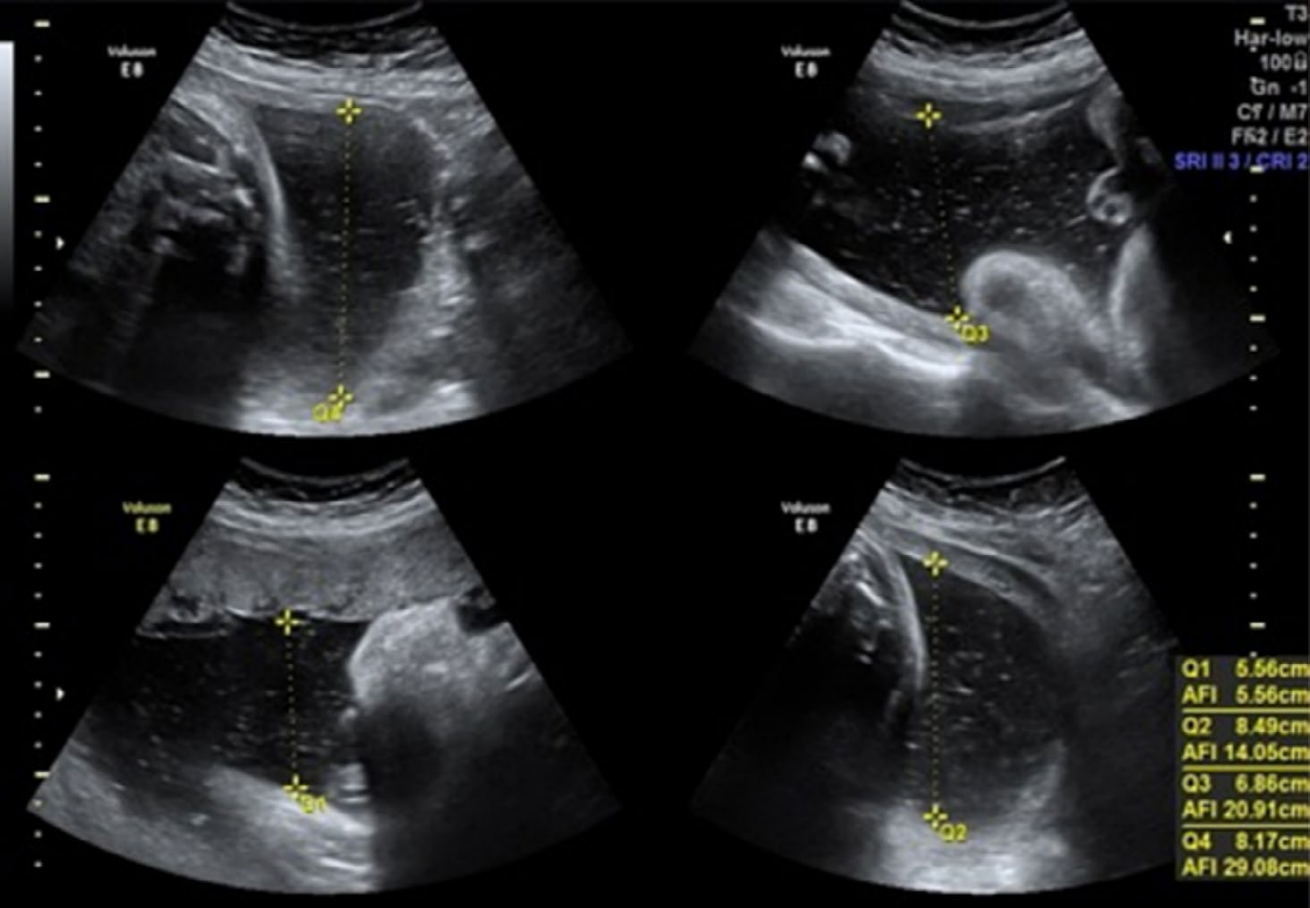
The four quadrants of amniotic fluid index (AFI).

A 27‐year‐old Caucasian female (Gravida 1, Para 0) with a spontaneous singleton pregnancy presented during the 27th week of gestation with presenting symptoms of shortness of breath and constipation. During the ultrasonographic examination, it was confirmed that a singleton gestation with normal fetal anatomy was present and the fetal growth was compatible with the gestational week. The amniotic fluid was elevated with an amniotic fluid index (AFI) measured at 29 cm (Figure 1). Her medical history was unremarkable, and she received only iron and multivitamin supplements. Her pregnancy was uneventful so far and the glucose tolerance test values were in normal ranges.

The patient was reassessed in a week and AFI was measured at 36 cm with a single deepest pocket (SDP) measured at 10.6 cm. After the diagnosis of severe hydramnios, as the risk for premature rupture of membranes (PROM) was increased, a decision for corticosteroid admission was made and a decompressive amniocentesis of 1 L of amniotic fluid was performed. Additionally, the amniotic fluid sample was sent for karyotype and biochemical investigation, measuring levels of chlorium, renin, and aldosterone.

### 1.2 Differential Diagnosis

Τhe biochemical results reported elevated levels of the renin, chlorium, and aldosterone in normal ranges (Table 1). Moreover, karyotype results were normal. Based on these results, the diagnosis of Bartter syndrome was suspected, and the sample was proceeded with for further genetic testing using Next Generation Sequencing (NGS).

**TABLE 1:** Levels of chlorium, renin, and aldosterone in the amniotic fluid.

Value	Result (units)	Normal range
Chlorium (Cl^−^)	116 mmol/L	98–106
Renin (direct)	706.0 mIU/mL	4.4–46.1 upright patient position
		2.8–39.9 sitting patient position
Aldosterone	15.1 ng/dL	4–31 upright patient position
		1–16 sitting patient position

## 2 Conclusion and Results

A week later, symptomatology reoccurred, and the patient visited the emergency department with shortness of breath. AFI was measured at 30 cm and SDP at 17 cm. Then, a second decompressive amniocentesis was performed, evacuating 1 L of amniotic fluid, resulting in the remission of the symptoms. However, 12 h after the second amniocentesis, the patient presented with PROM. The clinical examination revealed that the patient was in the active stage of labor with breech presentation. A decision for an emergency cesarean section was made, and a female neonate weighed 925 g was born at 28^+6^ weeks of gestation. The neonate was transferred to the neonatal intensive care unit and received special care from pediatric nephrologists.

The results of the genetic testing of the amniotic fluid are displayed in Table 2. A missense variant in the SLC12A1 gene in double heterozygosity was reported, a finding associated with Bartter syndrome type 1, with autosomal recessive gene inheritance.

**TABLE 2:** The results of the genetic testing of the amniotic fluid.

Gene	Nucleotide change	Amino acid changes	Outcome	Zygosity	Inheritance	Clinical significance
*SLC12A1* (NM_000338.3)	c.953C>A	p.Ala318Asp	Missense variant	Heterozygosity	Autosomal recessive	Unknown clinical significance
*SLC12A1* (NM_000338.3)	c.3247C>T	p.Pro1083Ser	Missense variant	Heterozygosity	Autosomal recessive	Unknown clinical significance

## 3 Discussion

Amniotic fluid (AFV) volume during pregnancy reflects fetal well‐being as both increased and decreased volume have been associated with perinatal adverse events. Polyhydramnios is defined as the abnormal increase of amniotic fluid volume, and this condition can be detected in approximately 1%–2% of all pregnancies [14]. Polyhydramnios is defined by the presence of a single deepest vertical pocket (DVP) of ≥ 8 cm or an amniotic fluid index (AFI) of ≥ 25 cm. Although the measurement of AFI, compared to DVP, has been associated with an increased diagnosis of reduced amniotic fluid and higher rates of medical intervention, it has not demonstrated improvements in perinatal outcomes. Nonetheless, both definitions are used interchangeably. Polyhydramnios is categorized as mild, moderate, or severe based on the amount of amniotic fluid present, with DVP measurements of 8–11, 12–15, and ≥ 16 cm, or AFI measurements of 25–29.9, 30–34.9, and ≥ 35 cm, respectively [15].

Polyhydramnios can be a final common pathway of several obstetric events, including chromosomal abnormalities, genetic syndromes, fetal structural malformations, fetal anemia, infections, placental abnormalities, and maternal diabetes mellitus. However, in the majority of cases (70%), no cause is identified, and the condition is classified as idiopathic [16]. The antenatal management of pregnant women diagnosed with idiopathic polyhydramnios poses a challenge for obstetricians and specialists in feto‐maternal medicine. Some studies indicate that mild cases of polyhydramnios do not necessitate antenatal fetal surveillance and support planning for spontaneous delivery. Conversely, other research advocates for heightened antenatal monitoring and delivery scheduling at 39 weeks due to the perceived elevated risk of adverse pregnancy outcomes linked to idiopathic polyhydramnios [14].

**FIGURE 2:**
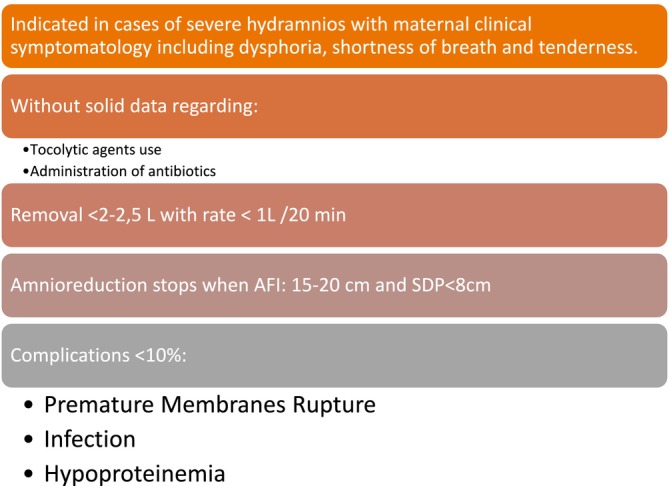
Amnioreduction basic points summary.

Amnioreduction (AR), previously a common approach in managing twin‐twin transfusion syndrome (TTTS) in monochorionic twin pregnancies before the advent of placental laser ablation, remains an option for relieving maternal symptoms and potentially prolonging the pregnancy duration. Despite several available data of literature on amnioreduction in TTTS, the published data concerning its application in singleton pregnancies remain scarce [17]. Regarding pregnancy outcomes following AR, previous studies have indicated a median gestational age at birth close to term (36–37 weeks). The benefits of AR in terms of prolonging pregnancy have been a topic of debate, alongside the reported increased likelihood of vaginal delivery and reduced incidence of uterine atony. Figure 2 depicts the basic points summary of amnioreduction. Perinatal outcomes are largely influenced by underlying fetal pathology [18]. AR is considered a safe procedure, associated with a low rate of short‐term peri‐procedural complications (preterm birth 4%–10%; premature rupture of membranes 0%–1%; fetal demise 0%–0.4%) [17].

Bartter's syndrome, initially documented by Bartter and colleagues in 1962 in order to describe a renal tubular disorder characterized by low serum levels of potassium (hypokalemia) and chloride (hypochloremia), metabolic alkalosis, and elevated levels of renin in the blood, while maintaining normal blood pressure [1]. The clinical symptoms of polyuria are caused by a decrease in the reabsorption of sodium chloride in the medullary thick ascending limb of the loop of Henle. This leads to a decrease in volume and an increase in the hormone aldosterone, resulting in low levels of potassium and increased excretion of potassium and hydrogen ions in the urine [1, 2].

Several mutations affecting genes responsible for regulating transporter production or integration have been identified. Bartter syndrome is classified into categories I to V based on symptom severity and clinical presentation [3, 4, 5, 6]. Types I to V manifest during fetal and neonatal stages, leading to excessive amniotic fluid volume during pregnancy and early delivery [19, 20]. Type I is associated with a mutation in the SLC12A2 gene, impairing the Na–K–2Cl symporter function. Conversely, type II is linked to a mutation in the ROMK/KCNJ1 gene, affecting the potassium channel in the thick ascending limb. Type III presents later in life with milder symptoms compared to types I and II, attributed to CLC‐Kb chloride channel inactivation. Neonatal Type IV occurs shortly after birth, associated with genetic changes in the ClC‐Ka and ClC‐Kb chloride channels, often leading to sensorineural deafness [7]. Gain‐of‐function mutations in the calcium‐sensing receptor (CaSR) are linked to hypocalcemia and hypomagnesemia development [8]. Moreover, an additional distinct subtype of the syndrome, considered as type V, is characterized by an autosomal dominant hypocalcemic hypercalciuria, and it is linked to gain‐of‐function mutations of CASR [6].

Our case involved a nucleotide missense c.953C>A(p.Ala318Asp) that is not reported in gnomAD [21], ClinVar [22], and Uniprot [23] databases, and in accordance with all bioinformatic tools, this mutation has a negative impact. However, the present missense has been reported in the literature as a variant of unknown significance in a patient with complex heterozygosity [17]. The nucleotide missense c.3247C>T(p.Pro1083Ser) is reported in the gnomAD database [21] (0.00000737%) without any cases of homozygosity being reported so far. In ClinVar [22] and Uniprot [23] databases, the above‐mentioned mutation has not been reported, and there are no cases published in the current literature so far.

Management of infants with types I, II, and IV during fetal and neonatal periods involves lifelong administration of fluid and electrolyte supplements. Non‐steroidal anti‐inflammatory drugs (NSAIDs) are also used to prevent excessive renal prostaglandin E2 production, with safety concerns regarding long‐term NSAID use, particularly in preterm infants.

A novel X‐linked variant of prenatal Bartter syndrome has recently been identified. This variant is associated with a transient illness caused by a mutation in the melanoma‐associated antigen D2 (MAGED2) gene [24]. Mutations in MAGED2 affect the expression and function of NKCC2 and NCC, sodium chloride cotransporters responsible for salt reabsorption in the distal renal tubule. Due to impaired renal salt transport via NKCC2 and NCC, transient Bartter syndrome is linked to high perinatal mortality and preterm birth [25]. Early onset of symptoms in affected individuals resembles a type I Bartter syndrome phenotype but with more severe manifestations, including earlier onset of polyhydramnios and labor. However, all symptoms spontaneously resolve in surviving infants [26].

Amniotic fluid levels of renin, chloride, and aldosterone should be monitored, as their elevation can result from mutations in the sodium chloride and potassium chloride co‐transporter gene (NKCC2) [26, 27]. Effective postnatal treatment, involving intensive medical intervention in the intensive care unit and leading to significant recovery of the newborn, underscores the importance of promptly identifying and treating Bartter syndrome. This case not only illustrates the complexities associated with acute respiratory distress syndrome (ARDS) but also emphasizes the potential for favorable outcomes with timely and effective medical intervention. Thus, it underscores the importance of vigilant prenatal evaluation of polyhydramnios, enabling early detection and implementation of management strategies that can profoundly influence outcomes for affected newborns [26, 27].

This case report illustrates the intricate and challenging process of diagnosing Bartter syndrome prenatally. During intrauterine development, excessive accumulation of amniotic fluid represents the main clinical manifestation of the disease. Amnioreduction accompanied by biochemical analysis focusing on aldosterone, renin, and chloride levels plays a pivotal role in the diagnostic process. The identification of elevated levels of these compounds in the amniotic fluid can significantly guide the diagnostic journey, allowing for closer monitoring of the pregnancy and the development of a tailored management plan.

## Author Contributions

**Athina A. Samara:** data curation, investigation, writing – original draft. **Paschalis Mousios:** investigation, methodology, writing – original draft. **Paraskevas Perros:** investigation, writing – original draft. **Antonios Koutras:** methodology, writing – review and editing. **Emmanouil Manolakos:** methodology, resources. **Eleftherios Anastasakis:** investigation, methodology. **Chara Skentou:** investigation, writing – review and editing. **Antonios Garas:** methodology, supervision, writing – review and editing. **Konstantinos Dafopoulos:** methodology, writing – review and editing. **Sotirios Sotiriou:** conceptualization, methodology, supervision, writing – review and editing.

## Ethics Statement

The study was conducted according to the guidelines of the Declaration of Helsinki. Institutional Review Board approval was omitted as there were no experimental or therapeutic protocols applied.

## Consent

Written informed consent has been obtained from the patient's parents to publish this paper.

## Conflicts of Interest

The authors declare no conflicts of interest.

## Data Availability

Data are available upon reasonable request.
